# Draft Genome Sequence of the First Pathogenic *Leptospira* Isolates from Ecuador

**DOI:** 10.1128/genomeA.00271-16

**Published:** 2016-05-05

**Authors:** Veronica Barragan, Jason W. Sahl, Kristin Wiggins, Jorge Chiriboga, Ana Salinas, Nancy E. Cantos, Mariana N. Loor, Bertha I. Intriago, Melba Morales, Gabriel Trueba, Talima Pearson

**Affiliations:** a The Center for Microbial Genetics and Genomics, Northern Arizona University, Flagstaff, Arizona, USA; b Instituto de Microbiologia, Colegio de Ciencias Biologicas y Ambientales, Universidad San Francisco de Quito, Quito, Ecuador; c Translational Genomics Research Institute, Flagstaff, Arizona, USA; d Ministerio de Salud Pública, Portoviejo, Ecuador

## Abstract

Pathogenic *Leptospira* spp. cause leptospirosis upon contact with mucosa through wounds or ingestion, leading to headaches, fever, jaundice, kidney or liver failure, or death in about 1.3 million people each year. Here, we present the draft genomes of one *L. santarosai* isolate and two *L. interrogans* isolates from Ecuador*.*

## GENOME ANNOUNCEMENT

Leptospirosis is a zoonotic disease with worldwide distribution (1), responsible for 1.03 million human cases per year around the world (2). Leptospirosis infection occurs by direct contact with urine or flesh from infected animals or by contact with contaminated soil or water (3). Ten out of 22 *Leptospira* spp. are classified in the “pathogenic clade” of the genus: *L. alexanderi*, *L. weilii*, *L. borgpetersenii*, *L. santarosai*, *L. kmetyi*, *L. alstonii*, *L. interrogans*, *L. kirschneri*, *L. mayottensis*, and *L. noguchii* (4). Most of these have been reported to cause high human morbidity. To date, 334 whole genome *Leptospira* sequences have been published, from which most of them (65%) belong to *L. interrogans*, followed by *L. santarosai* (8%), *L. kirschneri* (8%), and *L. borgpetersenii* (6%) (5).

Here, we announce the first three *Leptospira* whole-genome sequences from Ecuador. These isolates are from human blood collected in 2014 from Portoviejo, Ecuador (isolate C216), and cow urine (isolates ZV013 and ZV016) from Portoviejo collected in 2014. Isolates were obtained by culturing human blood (isolate C216) and cow urine (isolates ZV013, ZV016) in EMJH culture media. Research on human samples was approved by the Northern Arizona University Institutional Review Board (482212-1). Dual-indexed Illumina MiSeq libraries were prepared from genomic DNA as described in Keim et al. (6). Genome assembly was performed by using SPAdes version 3.60 (7). Comparative analysis of the 16S rRNA gene identified isolate C216 as *L. santarosai*, and isolates ZV013 and ZV016 as *L. interrogans*. A detailed list of genome assembly details is shown in Table 1. We used Prokka software (8) to annotate the genome and determine the total number of coding sequences, tRNAs, and rRNAs.

**TABLE 1:** Three *Leptospira* sp. genomes released to NCBI

Isolate ID	Accession no.	Source	Genome size (bp)	No. of contigs	No. of CDSs^a^	No. of tRNAs	No. of rRNAs
C216	LSSR00000000	Human sera	3,983,958	95	3,547	37	1
ZV013	LSSQ00000000	Cow urine	4,414,224	87	3,586	38	1
ZV016	LSSS00000000	Cow urine	4,416,860	117	3,590	38	1

^a^ CDSs, coding sequences.

### Nucleotide sequence accession numbers.

All three genomes have been deposited in GenBank under the accession numbers listed in Table 1. The versions in this paper are the first versions.
